# Smad7 Enhances TGF-β-Induced Transcription of c-Jun and HDAC6 Promoting Invasion of Prostate Cancer Cells

**DOI:** 10.1016/j.isci.2020.101470

**Published:** 2020-09-03

**Authors:** Noopur Thakur, Anahita Hamidi, Jie Song, Susumu Itoh, Anders Bergh, Carl-Henrik Heldin, Maréne Landström

**Affiliations:** 1 Ludwig Institute for Cancer Research, Ltd., Science for Life Laboratory, Uppsala University, Box 595, 751 24 Uppsala, Sweden; 2 Department of Medical Biochemistry and Microbiology, Science for Life Laboratory, Uppsala University, Box 582, 751 23 Uppsala, Sweden; 3 Department of Medical Biosciences, Umeå University, 901 87 Umeå, Sweden; 4 Laboratory of Biochemistry, Showa Pharmaceutical University, Tokyo 194-8543, Japan

**Keywords:** Molecular Biology, Cell Biology, Cancer

## Abstract

Transforming growth factor β (TGF-β) enhances migration and invasion of cancer cells, causing life-threatening metastasis. Smad7 expression is induced by TGF-β to control TGF-β signaling in a negative feedback manner. Here we report an additional function of Smad7, i.e., to enhance TGF-β induction of *c-Jun* and *HDAC6* via binding to their regulatory regions, promoting migration and invasion of prostate cancer cells. Lysine 102 in Smad7 is crucial for binding to specific consensus sites in *c-Jun* and *HDAC6,* even when endogenous Smad2, 3, and 4 were silenced by siRNA. A correlation between the mRNA expression of *Smad7* and *HDAC6*, *Smad7* and *c-Jun*, and *c-Jun* and *HDAC6* was found in public databases from analyses of prostate cancer tissues. High expression of Smad7, HDAC6, and c-Jun correlated with poor prognosis for patients with prostate cancer. The knowledge that Smad7 can activate transcription of proinvasive genes leading to prostate cancer progression provides clinically relevant information.

## Highlights

• TGF-β promotes prostate cancer cell migration and invasion, via Smad7
• Lysine 102 in Smad7 binds to DNA in regulatory regions of *HDAC6* and *c-Jun*
• Smad7 regulates expression of HDAC6 and c-Jun in prostate cancer in response to TGF-β
• High levels of Smad7, c-Jun, and HDAC6 are found in aggressive prostate cancer tissues

## Graphical Abstract

**Figure:**
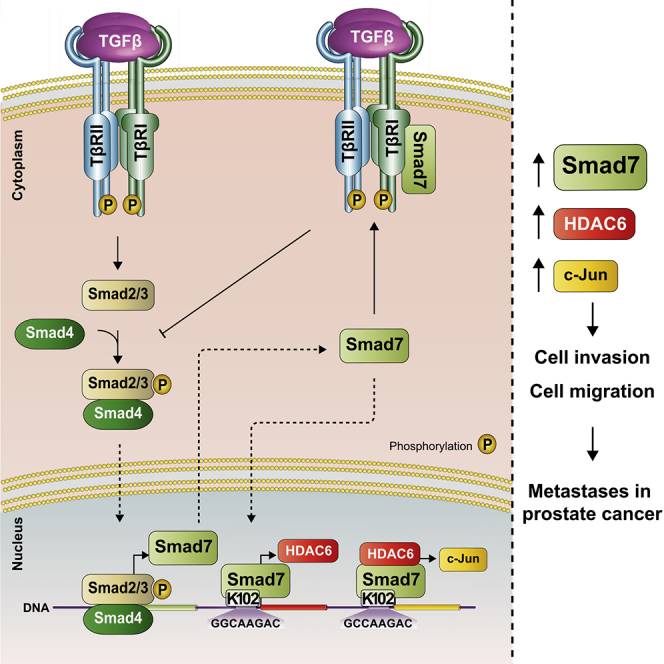
For a Figure360 author presentation of this figure, see https://doi.org/10.1016/j.isci.2020.101470.

## Introduction

TGF-β is a pleiotropic cytokine that regulates cellular responses, such as proliferation and migration, during embryonal development and tissue homeostasis; in addition, TGF-β signaling is perturbed in diseases, including tumorigenesis (Bierie and Moses, 2006; Derynck and Akhurst, 2007; Heldin and Moustakas, 2016; Massague, 2012). TGF-β signals through type I (TβRI) and type II (TβRII) serine/threonine kinase receptors leading to phosphorylation of R-Smads (Smad2 and Smad3), which form complexes with Smad4 that are translocated to the nucleus to regulate transcription of genes. Smad7, an inhibitory (I)-Smad, has been shown to inhibit TGF-β signaling by competing with R-Smads for receptor binding and by recruitment of ubiquitin ligases targeting the receptors for degradation and phosphatases for dephosphorylating and deactivating the receptors (Hanyu et al., 2001; Hayashi et al., 1997; Kwon et al., 2013; Morikawa et al., 2016; Shi and Massague, 2003). In addition, Smad7 binds to the Smad-binding elements (SBEs) of the *PAI-1* gene promoter and prevents the binding of R-Smads, thereby inhibiting the induction of PAI-1 by TGF-β (Zhang et al., 2007).

TGF-β also activates non-Smad signaling pathways (Edlund et al., 2003), in which the tumor necrosis factor (TNF) receptor-associated factor 6 (TRAF6) is crucial (Gudey et al., 2014; Hamidi et al., 2017; Mu et al., 2011; Sorrentino et al., 2008; Yamashita et al., 2008). In non-Smad signaling pathways, Smad7 acts as an adaptor bringing together TβRI and TRAF6 with other signaling components (Mu et al., 2012). Moreover, Smad7 also mediates cross talk between TGF-β and certain other signaling pathways; for instance, its expression is induced by γ-interferon/STAT, TNF-α/NF-κB, and epidermal growth factor (Afrakhte et al., 1998; Massague, 2008). The C-terminal region of Smad7 has a conserved Mad homology 2 (MH2) domain but lacks the SXS motif, which in R-Smads is phosphorylated by TβRI. The N-terminal MH1 domain, which is involved in the DNA binding of Smad3 and 4, is only partially conserved in Smad7 (Aragon et al., 2019; Heldin et al., 1997).

Prostate cancer is the most common cancer form and one of the leading causes of cancer-related deaths in men (Velonas et al., 2013). In the clinical situation, the diagnosis and prognosis are based on the histopathological grading by using the Gleason score, measurement of the prostate specific antigen (PSA) in blood and clinical staging of prostate cancer (Falzarano et al., 2015; Partin et al., 2002). Despite recent improvements, the existing treatment options for patients with recurrent disease are still limited (Nevo et al., 2019). It is therefore necessary to develop novel methods and biomarkers to predict prognosis and to design novel strategies for treatment of recurrent disease.

TGF-β has opposing effects on normal and malignant prostate cells (Danielpour, 2005). In normal prostate, TGF-β inhibits cell proliferation and regulates cell differentiation of epithelial cells, induces apoptosis, and maintains dormancy of prostate stem cells (Danielpour, 1999; Ricciardelli et al., 2005; Salm et al., 2005). During prostate cancer progression, increased TGF-β expression in stroma and epithelium has been reported (Shariat et al., 2004; Stravodimos et al., 2000), whereas loss of TGF-β receptors in malignant tissues protects them from anti-proliferative and pro-apoptotic effects of TGF-β (Kim et al., 1998). Recent studies have demonstrated that disruption of TGF-β signaling by introduction of a dominant negative TβRII in the prostate epithelium of a preclinical adenocarcinoma mouse model leads to accelerated tumor growth (Pu et al., 2017). The increased TGF-β levels in advanced prostate cancer induce stromal expansion, fibroblast-myofibroblast transdifferentiation, angiogenesis, extracellular matrix remodeling, epithelial-mesenchymal transition (EMT), immune suppression, and metastatic spread (Ahel et al., 2019; Assinder et al., 2009; Lee et al., 2000; Wikstrom et al., 1998). Aberrant TGF-β signaling has been observed in a preclinical prostate cancer model, in which the tumor suppressor PTEN is silenced specifically in the prostate, leading to premalignant alterations of the prostate epithelium. In this model, enhanced activation of the PI3K pathway and increased levels of Smad4 protein were observed, whereas reduced Smad4 expression correlated with metastatic tumor progression, supporting a role of Smad4 acting as a tumor suppressor in prostate cancer (Ding et al., 2011). The report from Ding et al. also identified two genes as being crucial for prostate cancer progression, i.e., cyclin D1 and SPP1 (Ding et al., 2011). Based on this finding it is important to achieve further knowledge about the role and function of aberrant TGF-β signaling in prostate cancer and to identify potential novel treatment strategies for aggressive prostate cancer.

The aim of the present study was to explore the possibility that Smad7 has a role as a transcription factor in the nucleus. We found that Smad7 positively regulates the expression of *HDAC6* and *c-Jun* by binding to their regulatory regions in a TGF-β-dependent manner.

## Results

### TGF-β-Induced Expression of c-Jun and HDAC6 mRNA and Protein Is Dependent on Smad7

**Figure 1:**
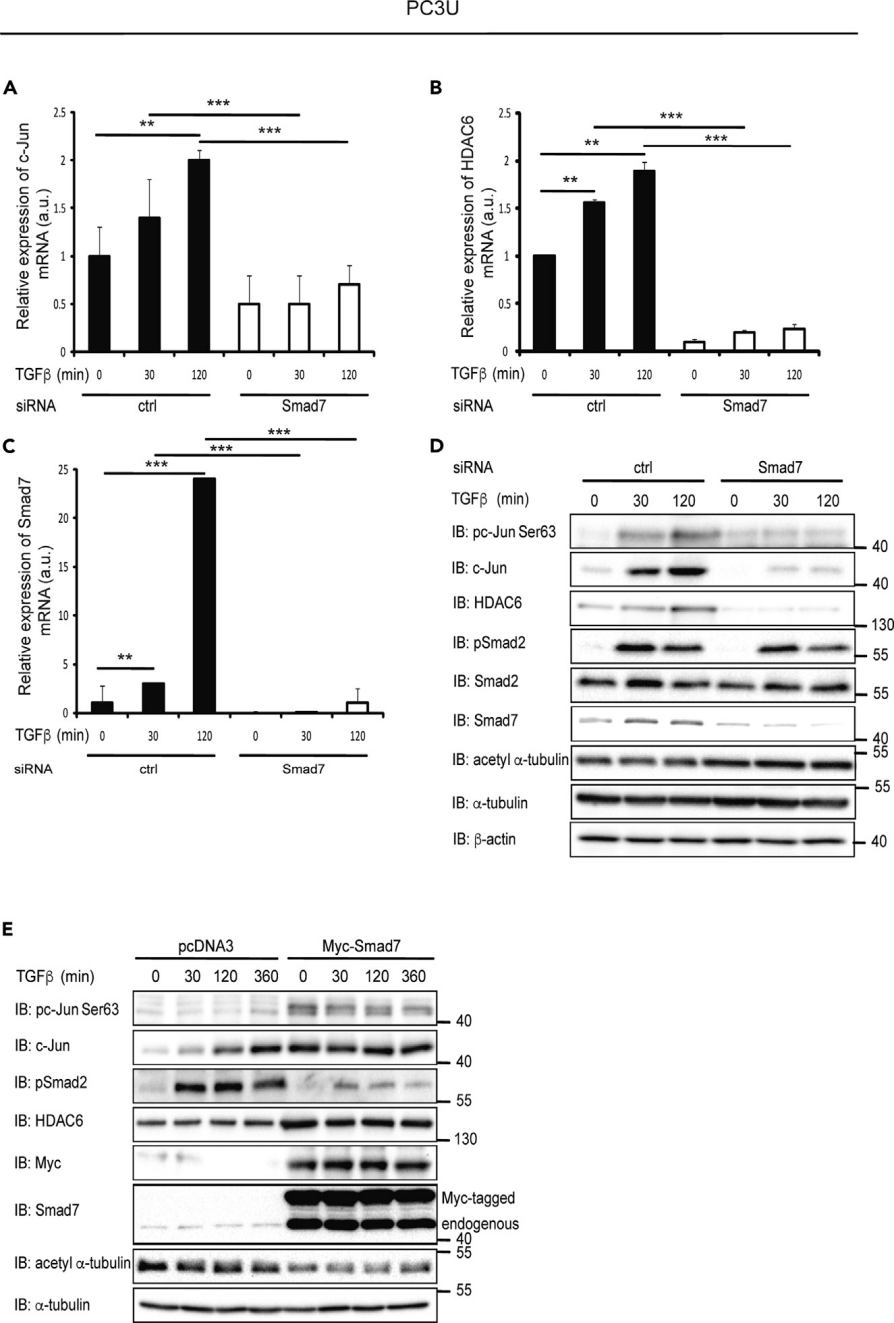
TGF-β-Induced Expression of c-Jun and HDAC6 mRNA and Protein Is Dependent on Smad7 (A–C) PC3U cells transfected with control (ctrl) or Smad7 siRNA and stimulated with TGF-β, or not, were lysed and subjected to RT-PCR using c-Jun (A), HDAC6 (B), or Smad7 (C) primers. Graphs are means ± SEM from three independent experiments. One-way ANOVA was used as the statistical test. ∗∗p < 0.01, ∗∗∗p < 0.001. (D) PC3U cells transfected with ctrl or Smad7 siRNA and stimulated or not with TGF-β were lysed and subjected to immunoblotting (IB). (E) Lysates from PC3U cells transfected with pcDNA3 or Myc-Smad7 plasmids and stimulated with TGF-β, or not, were subjected to immunoblotting. See also Figures S1 and S2.

Stimulation of PC3U cells with TGF-β1 led to induction of both c-Jun and HDAC6 (Figure 1). To investigate the importance of Smad7 for HDAC6 and c-Jun expression, Smad7 was knocked down by siRNA in the prostate cancer cell lines PC3U, LNCaP, and DU145; this led to a decreased expression of both *c-Jun* and *HDAC6* mRNA (Figures 1A–1C, S1A, and S2A) and protein (Figures 1D, S1B, and S2B). Overexpression of Myc-Smad7 by transfection in PC3U cells led to an increase in the expression of total c-Jun and HDAC6 (Figure 1E). Our results thus support the notion that Smad7 not only negatively regulates TGF-β signaling (Budi et al., 2017) but also positively regulates the expression of certain genes, including *c-Jun* and *HDAC6*.

### Smad7 Binds to a 5′-GGCA-3′element in *HDAC6* and *c-Jun* Regulatory Regions

**Figure 2:**
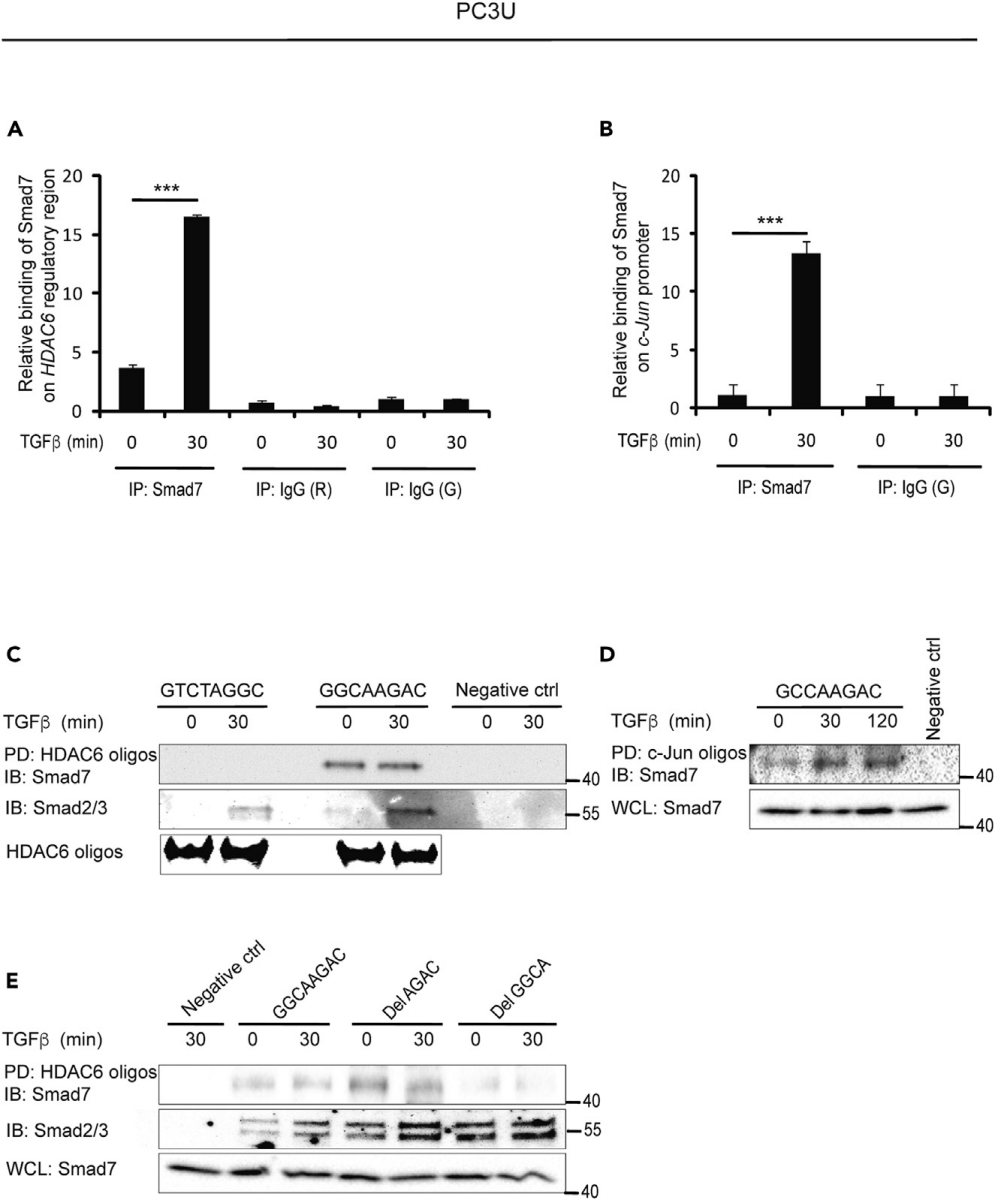
Smad7 Binds to a 5′-GGCA-3′Element in *HDAC6* and 5′-GCCA-3′Element in *c-Jun* Regulatory Regions (A and B) PC3U cells stimulated or not with TGF-β were lysed and ChIP assay was performed using a Smad7 antibody, or control rabbit (R) or goat (G) IgG, for immunoprecipitation, and primers recognizing *HDAC6* regulatory region (A) and *c-Jun* promoter (B). Graphs are means ± SEM from three independent experiments. One-way ANOVA was used as the statistical test. ∗∗∗p < 0.001. (C and D) PC3U cells were stimulated or not with TGF-β and DNAP was performed by pull-down (PD) of biotinylated GTCTAGGC, GGCAAGAC (C) or GCCAAGAC (D) oligos by streptavidin-agarose, followed by immunoblotting (IB) for Smad7. No oligo was added in the PD step for the negative ctrl. The filter was reblotted for Smad2/3. HDAC6 oligos were analyzed by agarose electrophoresis gel (C). (E) PC3U cells were stimulated, or not, with TGF-β and DNAP was performed by PD using biotinylated GGCAAGAC, Del AGAC, or Del GGCA HDAC6 oligos, followed by IB for Smad7. No oligo was added in the PD step for the negative ctrl. The filter was reblotted for Smad2/3. IB for Smad7 was performed on whole-cell lysates (WCL) as a control of the input levels.

Chromatin immunoprecipitation (ChIP) was performed using different regions of the *c-Jun* and *HDAC6* promoters and regulatory regions to explore the possibility that Smad7 binds directly to sequences in the *c-Jun* and *HDAC6* genes. Regions of about 250 base pairs (bp) in intron 6 of *HDAC6* and in the *c-Jun* promoter were found to be Smad7-binding sites (Figures 2A and 2B). In order to narrow down the potential binding site for Smad7, we analyzed the regulatory regions of the *HDAC6* and *c-Jun* genes for similarities. Two candidate binding sites were found in the regulatory regions of *HDAC6* and one in *c-Jun*. To investigate whether they bound Smad7, DNA affinity precipitation (DNAP) was performed with 52-bp biotinylated oligos containing GGCAAGAC or GTCTAGGC sequences in the *HDAC6* regulatory region and the GCCAAGAC sequence in the *c-Jun* promoter; Smad7 was found to bind to GGCAAGAC in the regulatory region of *HDAC6* and to GCCAAGAC in the *c-Jun* promoter (Figures 2C and 2D). These sequences contain a 5′-AGAC-3′ motif, which is a known binding site for Smad3 and 4. In order to investigate whether the 5′-AGAC-3′ motif is needed for binding of Smad7, we designed deletion oligonucleotides lacking GGCA or AGAC and determined the binding of Smad7. Smad7 bound to the oligonucleotide lacking the 5′-AGAC-3′ element, but not to the oligonucleotide lacking 5′-GGCA-3′, indicating that the GGCA sequence, but not the AGAC sequence, is needed for the binding of Smad7 (Figure 2E).

### Smad2, 3, and 4 Are Not Required for Smad7 to Bind to DNA

**Figure 3:**
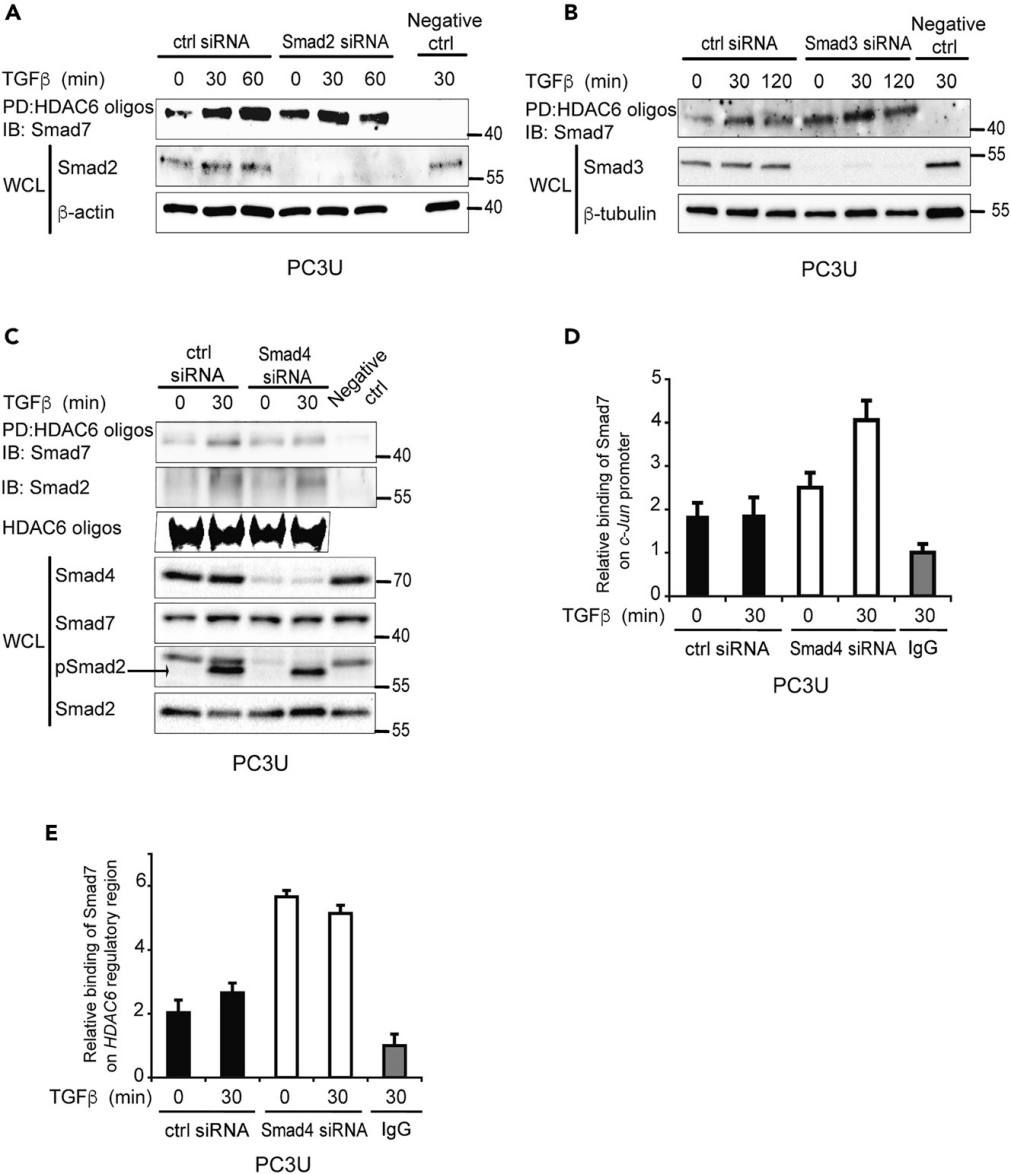
Smad2, 3, and 4 Are Not Required for Smad7 to Bind to DNA (A–C) Lysates from PC3U cells transfected with ctrl or Smad2 (A), Smad3 (B), or Smad4 (C) siRNA, and treated or not with TGF-β, were subjected to DNAP assay using biotinylated HDAC6 oligos for PD and Smad7 antibody for IB. DNAP filter was reblotted with Smad2 antibody (C) No oligo was added in the PD step for the negative ctrl. Corresponding WCLs were subjected to IB for Smad2 (A), Smad3 (B), Smad4, Smad2, pSmad2 (C). IB for Smad7 was performed on WCL as a control of the input levels (C). Biotinylated HDAC6 oligos with GGCAAGAC binding sites were run on an agarose electrophoresis gel (C). (D and E) Lysates from PC3U cells transfected with ctrl or Smad4 siRNA, and treated or not with TGF-β, were subjected to ChIP with Smad7 or IgG ctrl antibody and RT-PCR with primers recognizing *c-Jun* promoter (D) or *HDAC6* regulatory region (E). Graphs are means ± SEM from three independent experiments. See also Figure S3.

As a Smad2/3/4-binding site was located adjacent to the Smad7-binding site in the *c-Jun* and *HDAC6* regulatory regions, it is possible that the binding of Smad7 to DNA depends on Smad2, 3, or 4. To explore this possibility, DNAP was performed on the lysates from PC3U cells transfected with control siRNA and siRNA for Smad2, 3, or 4. Smad7 was found to bind to the *HDAC6* regulatory region even in the absence of Smad2, 3, or 4 (Figures 3A–3C). ChIP assays using PC3U cells transfected with control, Smad2, Smad3 (Figures S3A and S3B), or Smad4 siRNA (Figures 3D and 3E) also showed that binding of Smad7 on the *c-Jun* and *HDAC6* regulatory regions does not require Smad2, 3, or 4.

### K102 of Smad7 Is Important for Binding to the *HDAC6* and *c-Jun* Regulatory Regions

**Figure 4:**
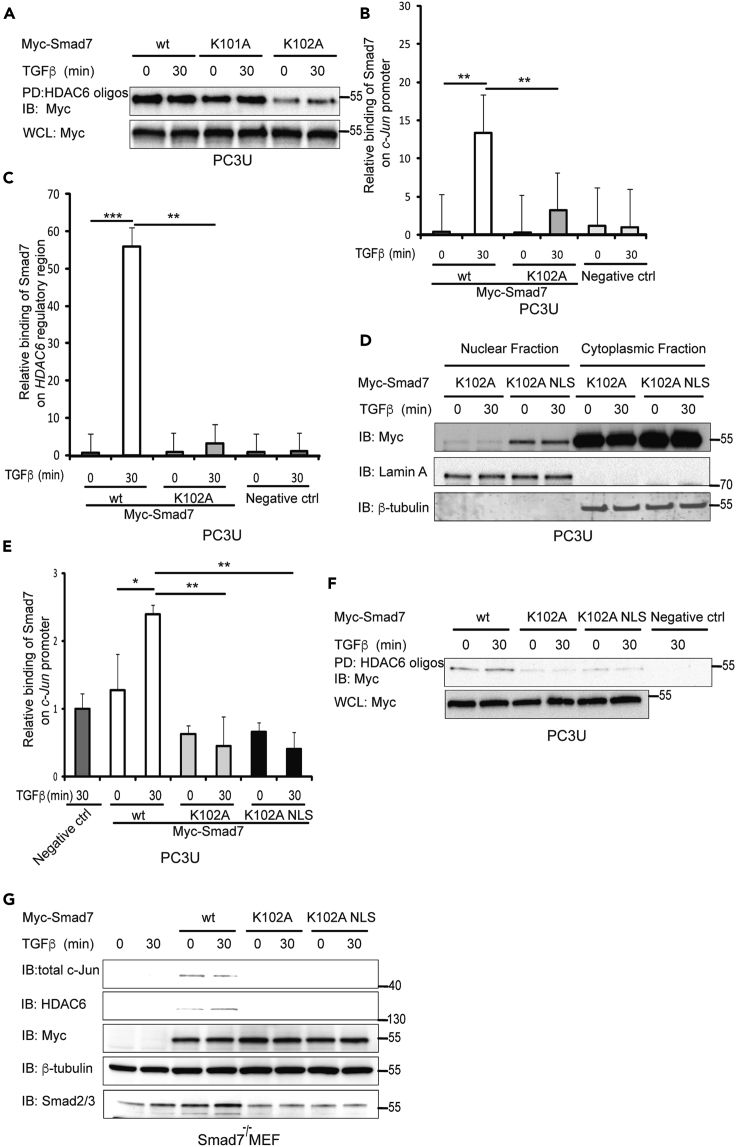
Lysine102 (K102) of Smad7 Is Important for Binding to the *HDAC6* and *c-Jun* Regulatory Regions (A) Cells transfected with wt Smad7, K101A or K102A mutant Smad7, and treated with TGF-β, were lysed and DNAP assay was performed on lysates using biotinylated HDAC6 oligos for PD and Myc antibody for IB. Corresponding WCLs were IB for Myc as a control for equal transfection. (B and C) Cells were transfected with wt or K102A mutant Smad7 plasmids and treated or not with TGF-β and lysed. Cell lysates were subjected to ChIP using Smad7 or ctrl IgG antibodies for IP and primers recognizing *c-Jun* promoter (B) and *HDAC6* regulatory region (C). Graphs are means ± SEM from three independent experiments. One-way ANOVA was used as the statistical test. ∗∗p < 0.01, ∗∗∗p < 0.001. (D) PC3U cells transfected with K102A or K102A NLS mutants of Myc-Smad7 were treated or not with TGF-β and lysed. Lysates were subjected to cytoplasmic-nuclear fractionation followed by IB for Myc, Lamin A/C, and β-tubulin. (E) Lysates from PC3U cells transfected with wt, K102A or K102A NLS mutant Myc-Smad7 and treated with TGF-β, were subjected to ChIP assay by using Smad7 or IgG control antibody for IP and primers recognizing *c-Jun* promoter for RT-PCR. Graphs are means ± SEM from three independent experiments. One-way ANOVA was used as the statistical test. ∗p < 0.05, ∗∗p < 0.01. (F) Cells transfected with wt Smad7, K102A or K102A NLS mutants of Myc-Smad7, and treated with TGF-β, were lysed and DNAP assay was performed on lysates using biotinylated HDAC6 oligos for PD and Myc antibody for IB. Corresponding WCL were IB for Myc as a control for equal transfection. (G) Smad7^−/−^ MEFs transfected or not with wt Smad7, K102A or K102A NLS mutants of Myc-Smad7 plasmid and treated with TGF-β were lysed, and lysates were IB for c-Jun, HDAC6, Myc, β-tubulin, and Smad2/3.

In order to identify possible DNA-binding epitopes in Smad7, we focused on basic amino acid residues in the region that are homologous with the DNA-binding region of Smad4, i.e., lysine 101 (K101) and K102. Each of the lysine residues were mutated to alanine residues and DNAP was performed in cells transfected with the different mutants. Mutation of K101 did not affect the binding of Smad7 to the regulatory region of *HDAC6*, whereas mutation of K102 to alanine reduced the binding, suggesting that this particular residue contributes to the efficient binding of Smad7 to DNA (Figure 4A). ChIP was also performed using lysates from cells transfected with wt Smad7, as well as K102A mutant Smad7; the results confirmed that wt Smad7 bound the regulatory regions of *c-Jun* and *HDAC6* but the K102A mutant bound less efficiently (Figures 4B and 4C). As K102 is located in a putative nuclear localization signal (NLS) of Smad7, the K102A mutation may have disrupted the nuclear entry of mutant Smad7 and therefore decreased the binding to the *c-Jun* and *HDAC6* regulatory regions. In order to assure an efficient nuclear translocation of the Smad7 K102A mutant, we fused an NLS sequence to the mutant construct. Cell fractionation showed that K102A Smad7 mutant supplemented with an NLS localized to the nucleus (Figure 4D). ChIP was performed to further examine the DNA-binding abilities of wt, as well as K102A and K102A-NLS mutant Smad7 on the *c-Jun* promoter. A reduced binding to the *c-Jun* promoter was observed for the mutants (Figure 4E). Moreover, DNAP was performed using PC3U cells transfected with wt Smad7, and Smad7 K102A and K102A-NLS mutants. wt Smad7 bound to the *HDAC6* regulatory region, but the Smad7 K102A and K102A-NLS mutants did not (Figure 4F). Taken together with the results obtained by DNAP experiments, the results of the ChIP experiment showed that wt Smad7 bind to the promoter of *c-Jun*, whereas the K102A and K102A-NLS mutant Smad7 did not bind. To further elucidate the role of Smad7 in regulating the expression of c-Jun and HDAC6, the expressions of c-Jun and HDAC6 were determined by immunoblotting of lysates of Smad7^−/−^ MEFs transfected by wt Smad7, or the K102A or K102A-NLS Smad7 mutants (Itoh et al., 1998). The expression of both c-Jun and HDAC6 was rescued by the transfection of wt Smad7 but not by the transfection of the Smad7 mutants (Figure 4G), demonstrating that Smad7 regulates these genes by directly binding to their regulatory regions.

### Smad2/3 Binding to the *HDAC6* and *c-Jun* Regulatory Regions Depends on Smad7

**Figure 5:**
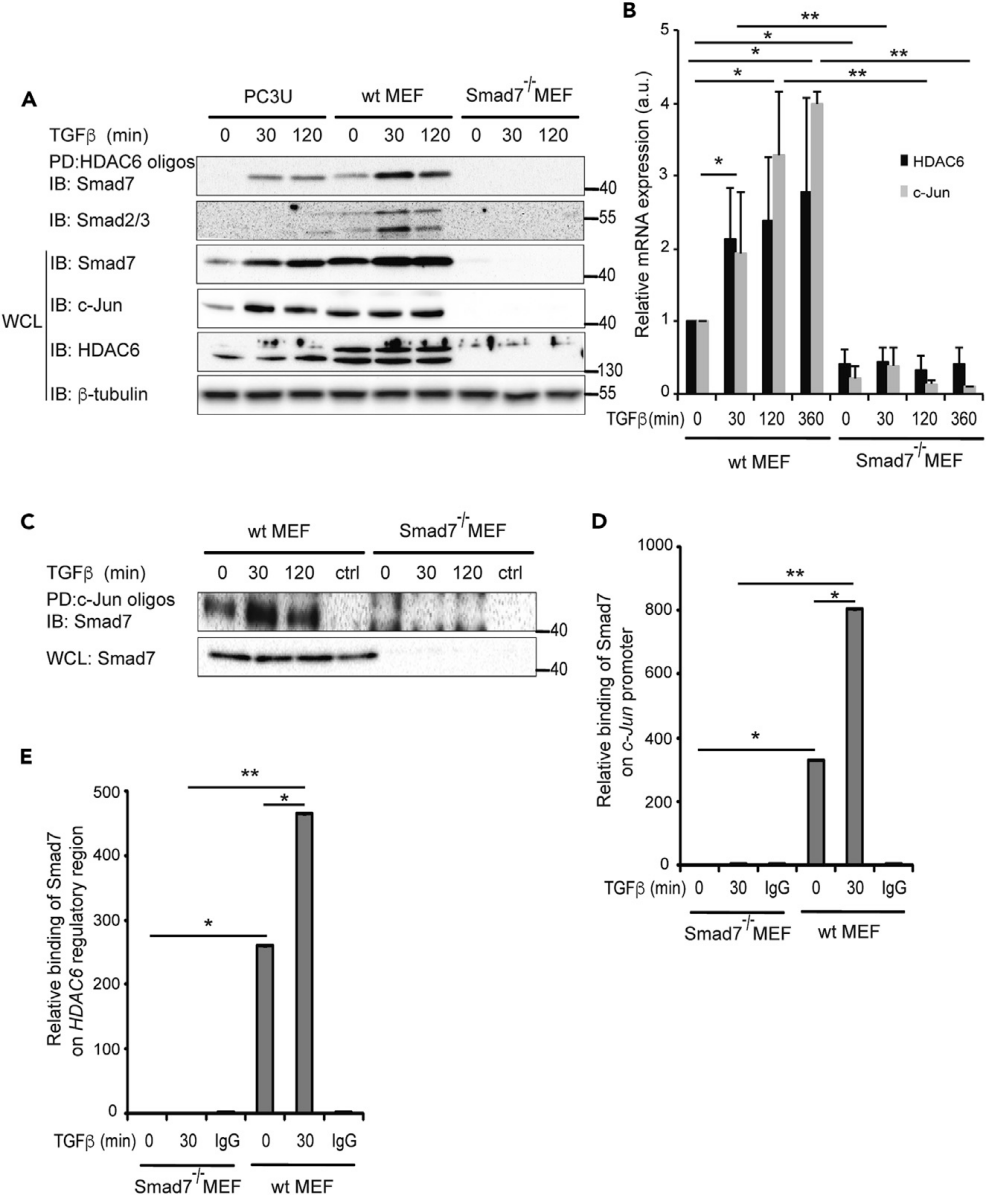
Smad2/3 Binding to the *HDAC6* and *c-Jun* Regulatory Regions Depends on Smad7 (A) PC3U, wt MEF cells, and Smad7^−/−^ MEF cells were treated with TGF-β and lysates of the cells subjected to DNAP by HDAC6 oligos, followed by IB for Smad7. The same filter was reblotted with a Smad2/3 antibody. The corresponding cell lysates were IB for Smad7, c-Jun, HDAC6, and β-tubulin. (B) After treatment with TGF-β, wt and Smad7^−/−^ MEF cells were lysed and mRNA expression of c-Jun and HDAC6 were measured by RT-PCR. (C) After treatment with TGF-β, wt and Smad7^−/−^ MEF cells were lysed and DNAP was performed by PD with c-Jun oligos and IB for Smad7. (D and E) wt and Smad7^−/−^ MEF cells treated or not with TGF-β were exposed to ChIP assay with a Smad7 antibody or ctrl IgG and analyzed with RT-PCR using primers for the *c-Jun* promoter (D) or *HDAC6* regulatory region (E). Graphs are means ± SEM from three independent experiments. One-way ANOVA was used as the statistical test. ∗p < 0.05, ∗∗p < 0.01.

In order to further investigate the binding of Smad7 to the regulatory regions of *HDAC6* and *c-Jun*, DNAP assays were performed using wt MEFs, as well as Smad7^−/−^ MEFs; PC3U cells were used for comparison. Smad7, as well as Smad2/3, from PC3U cells and wt MEFs bound to HDAC6 oligos (Figure 5A). Smad2/3 binding was lost in Smad7^−/−^ MEFs, suggesting that Smad7 is required for R-Smads to bind to the regulatory region of *HDAC6.* HDAC6 and c-Jun protein (Figure 5A) and c-Jun and HDAC6 mRNA (Figure 5B) expression was reduced significantly in Smad7^−/−^ MEFs, suggesting that Smad7 binding to the regulatory regions is required for the expression of both c-Jun and HDAC6. Smad7 from wt MEFs bound to c-Jun oligos, but as expected, no binding was observed in Smad7^−/−^ MEFs (Figure 5C). MEFs were also subjected to ChIP using a Smad7 antibody; Smad7 binding to the *c-Jun* and *HDAC6* regulatory regions was observed in wt MEFs but, as expected, not in Smad7^−/−^ MEFs (Figures 5D and 5E).

### Smad7 and HDAC6 Promote TGF-β-Induced Invasion and Migration

**Figure 6:**
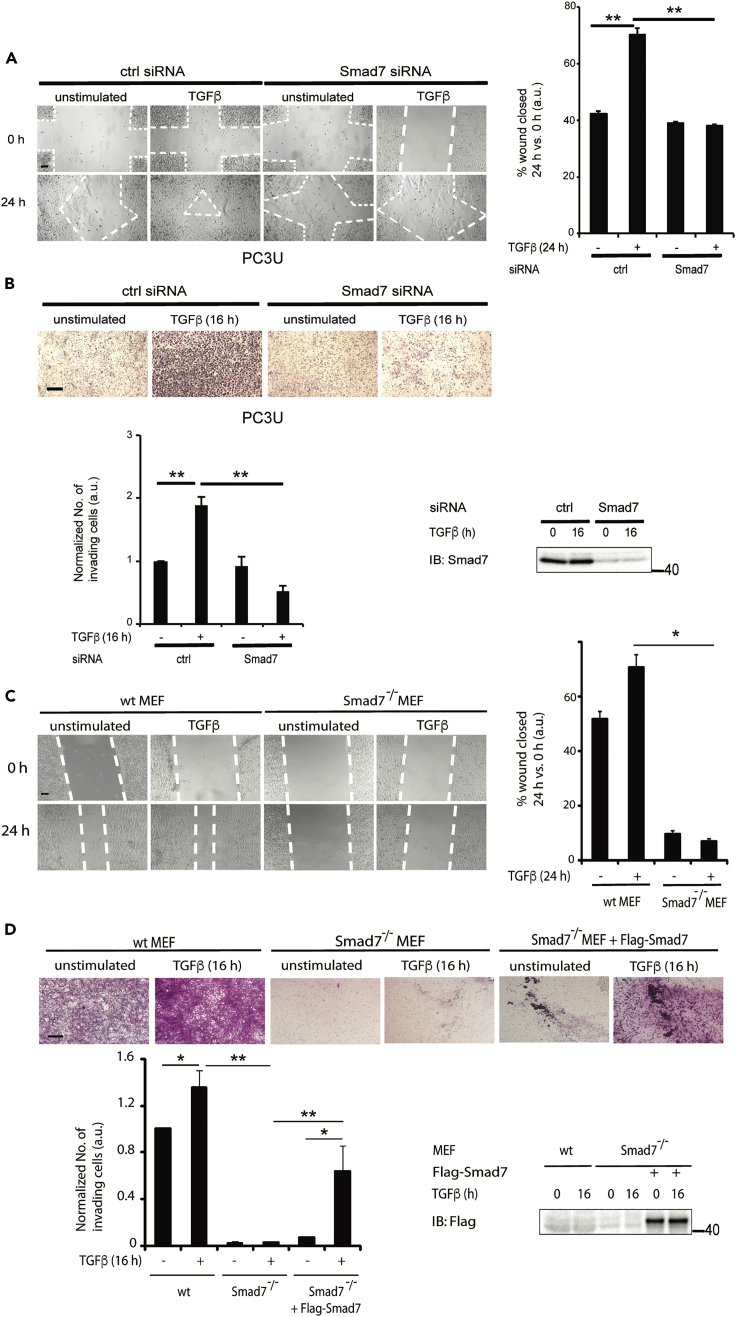
Smad7 and HDAC6 Promote TGF-β-Induced Invasion and Migration (A) PC3U cells transfected with ctrl or Smad7 siRNA, were stimulated, or not, with TGF-β in a cell culture wound healing assay, as described in Methods. (B) PC3U cells were subjected to an invasion assay for 16 h without or with TGF-β stimulation. Scale bar, 100 μM. Corresponding WCLs were exposed to IB with Smad7 antibody as a control. (C) wt and Smad7^−/−^ MEF cells were stimulated, or not, with TGF-β in a cell culture wound healing assay. (D) wt, Smad7^−/−^, and Smad7-retransfected Smad7^−/−^ MEF cells were subjected to an invasion assay for 16 h without or with TGF-β stimulation. Scale bar, 100 μM. Graphs are means ± SEM from three independent experiments. One-way ANOVA was used as the statistical test ∗p < 0.05, ∗∗p < 0.01.

To further validate a role for Smad7 in the regulation of TGF-β-induced migration and invasion, we investigated these responses in PC3U cells transfected with control or Smad7 siRNA. TGF-β-induced migration and invasion were abolished in cells in which Smad7 had been knocked down (Figures 6A and 6B), in line with our previous observations that Smad7 plays an important role for TGF-β-induced migration (Ekman et al., 2012). We further investigated these responses in wt and Smad7^−/−^ MEFs. We observed a complete loss of TGF-β-induced migration of Smad7^−/−^ MEFs in a cell culture scratch assay (Figure 6C). Moreover, TGF-β-induced invasion through a Matrigel-coated chamber was completely lost in Smad7^−/−^ MEFs but was regained upon transfection of Smad7 (Figure 6D). From these data, we conclude that Smad7 is required for TGF-β-induced migration and invasion of MEFs.

**Figure 7:**
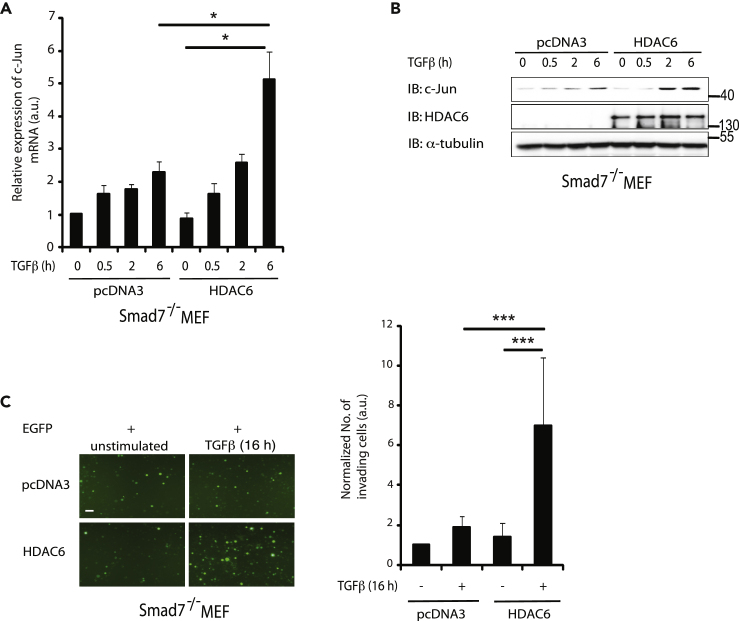
HDAC6 Acts Downstream of Smad7 to Promote c-Jun Expression and Invasion (A and B) Smad7^−/−^ cells transfected with pcDNA3 or HDAC6 plasmids and treated or not with TGF-β were lysed, and cell lysates were subjected to RT-PCR by using *c-Jun* primers (A) or IB for c-Jun, HDAC6, and α-tubulin (B). (C) Smad7^−/−^ cells transfected with EGFP and HDAC6 or pcDNA3 plasmids were stimulated or not with TGF-β and subjected to an invasion assay, as described in Methods. Scale bar, 100 μM. Bar graph are means ± SEM from three independent experiments. One-way ANOVA was used as the statistical test. ∗p < 0.05, ∗∗∗p < 0.001.

We have previously shown that c-Jun promotes invasion in prostate cancer cell (Thakur et al., 2014). Since Smad7 binds to the *HDAC6* regulatory region (Figure 2A) and has a role in cell migration and invasion in response to TGF-β (Figures 6A–6D), we investigated whether *c-Jun* mRNA and protein expression are affected by HDAC6 transfection in Smad7^−/−^ MEF cells. The TGF-β-induced increase in *c-Jun* mRNA (Figure 7A) and c-Jun protein (Figure 7B) was significantly higher in Smad7^−/−^ MEFs transfected with HDAC6 than in control Smad7^−/−^ MEFs. In order to further examine the role of HDAC6 in TGF-β-induced cell invasion, Smad7^−/−^ MEF cells were co-transfected with EGFP and HDAC6, or pcDNA3 as a control. Cells transfected with HDAC6, visualized by the fluorescent signal of EGFP, showed increased invasion compared with pcDNA3-transfected control cells (Figure 7C). These data demonstrate that HDAC6 exerts its role downstream of Smad7.

### HDAC6 Is Required for TGF-β-Induced c-Jun-Mediated Migration and Invasion

**Figure 8:**
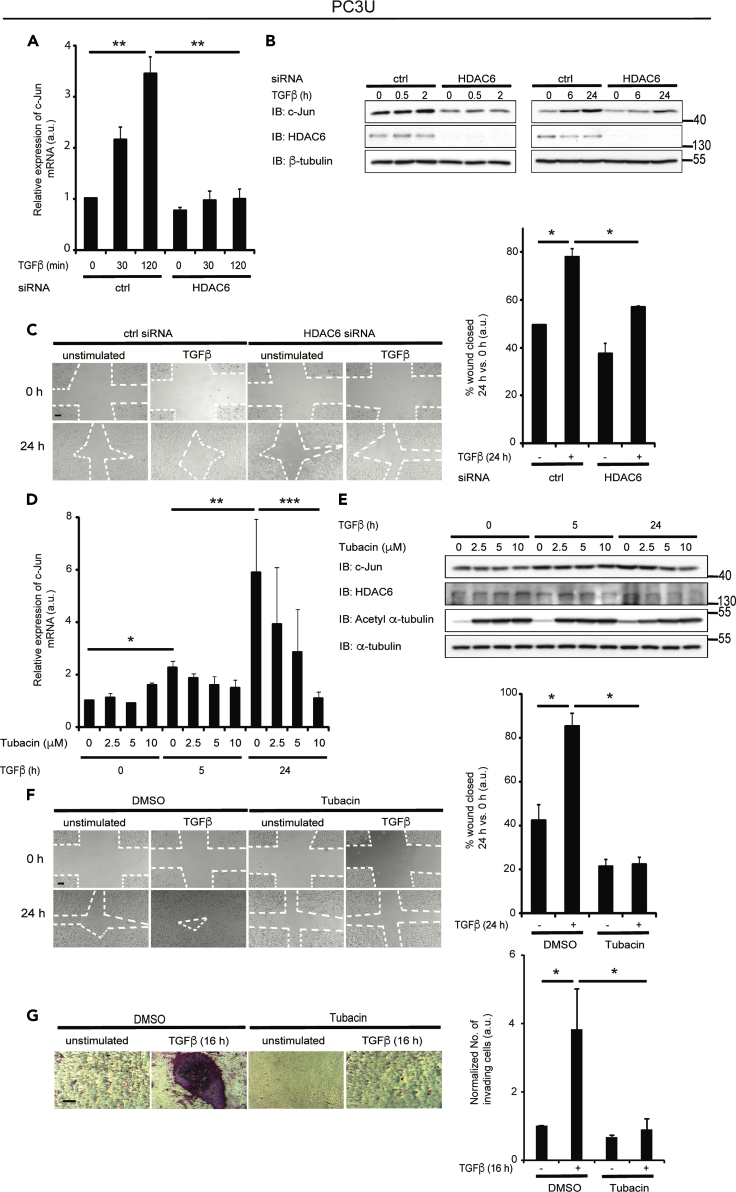
HDAC6 Is Required for TGF-β-Induced c-Jun-Mediated Migration and Invasion (A and B) PC3U cells transfected with control or HDAC6 siRNA and treated or not with TGF-β were lysed, and cell lysates were subjected to RT-PCR by using c-Jun primers (A), and IB for c-Jun, HDAC6, and β-tubulin (B). (C) PC3U cells transfected with ctrl or HDAC6 siRNA were stimulated or not with TGF-β in a scratch assay, as described in Methods. Scale bar, 100 μM. (D) PC3U cells were treated or not with 2.5, 5, and 10 μM tubacin prior to TGF-β stimulation, and mRNA expression of c-Jun was measured by RT-PCR. (E) The corresponding WCLs were subjected to IB using antibodies against c-Jun, HDAC6, acetyl α-tubulin, and α-tubulin. (F) After treatment with DMSO or 2.5 μM tubacin, PC3U cells were stimulated, or not, with TGF-β and subjected to a wound healing assay, as described in Methods. Scale bar, 100 μM. (G) PC3U cells were subjected to an invasion assay for 16 h without or with TGF-β stimulation and without or with tubacin. Scale bar, 100 μM. Bar graph are means ± SEM from three independent experiments. One-way ANOVA was used as the statistical test. ∗p < 0.05, ∗∗p < 0.01, and ∗∗∗p < 0.001.

We next investigated whether *c-Jun* mRNA and protein expression are dependent on HDAC6. HDAC6 knockdown by siRNA in PC3U cells resulted in decreased TGF-β-induced expression of *c-Jun* mRNA (Figure 8A) and c-Jun protein (Figure 8B). In a scratch assay, HDAC6 siRNA-transfected PC3U cells migrated slower than control siRNA-transfected cells in response to TGF-β (Figure 8C). To further investigate the role of HDAC6 in TGF-β-induced, c-Jun-dependent migration and invasion, PC3U cells were treated with increasing concentrations of tubacin, an HDAC6 inhibitor, added before TGF-β stimulation. TGF-β-induced mRNA expression of c-Jun was suppressed by tubacin treatment in a dose-dependent manner (Figure 8D), and the amount of c-Jun protein was decreased at high doses of tubacin after 24 h of TGF-β stimulation in PC3U (Figure 8E), as well as LNCaP (Figure S1C) and DU145 (Figure S2C) cells. PC3U cells treated with tubacin, or DMSO as a control, prior to TGF-β stimulation, were subjected to scratch (Figure 8F) and invasion (Figure 8G) assays. Treatment of cells with tubacin inhibited TGF-β-induced closure of the wound, whereas the wound closed in the control cell culture (Figure 8F). Similarly, TGF-β-induced invasion of PC3U cells was significantly decreased by tubacin treatment (Figure 8G). Two additional prostate cancer cells, LNCaP (Figure S1D) and DU145 (Figure S2D), also showed decreased TGF-β-induced invasion by tubacin treatment. These results suggest that HDAC6 is required for the TGF-β-induced c-Jun-mediated migration in prostate cancer cells.

### High Expression of *Smad7, c-Jun*, and *HDAC6* is Correlated with Poor Prognosis in Patients with Prostate Cancer

**Figure 9:**
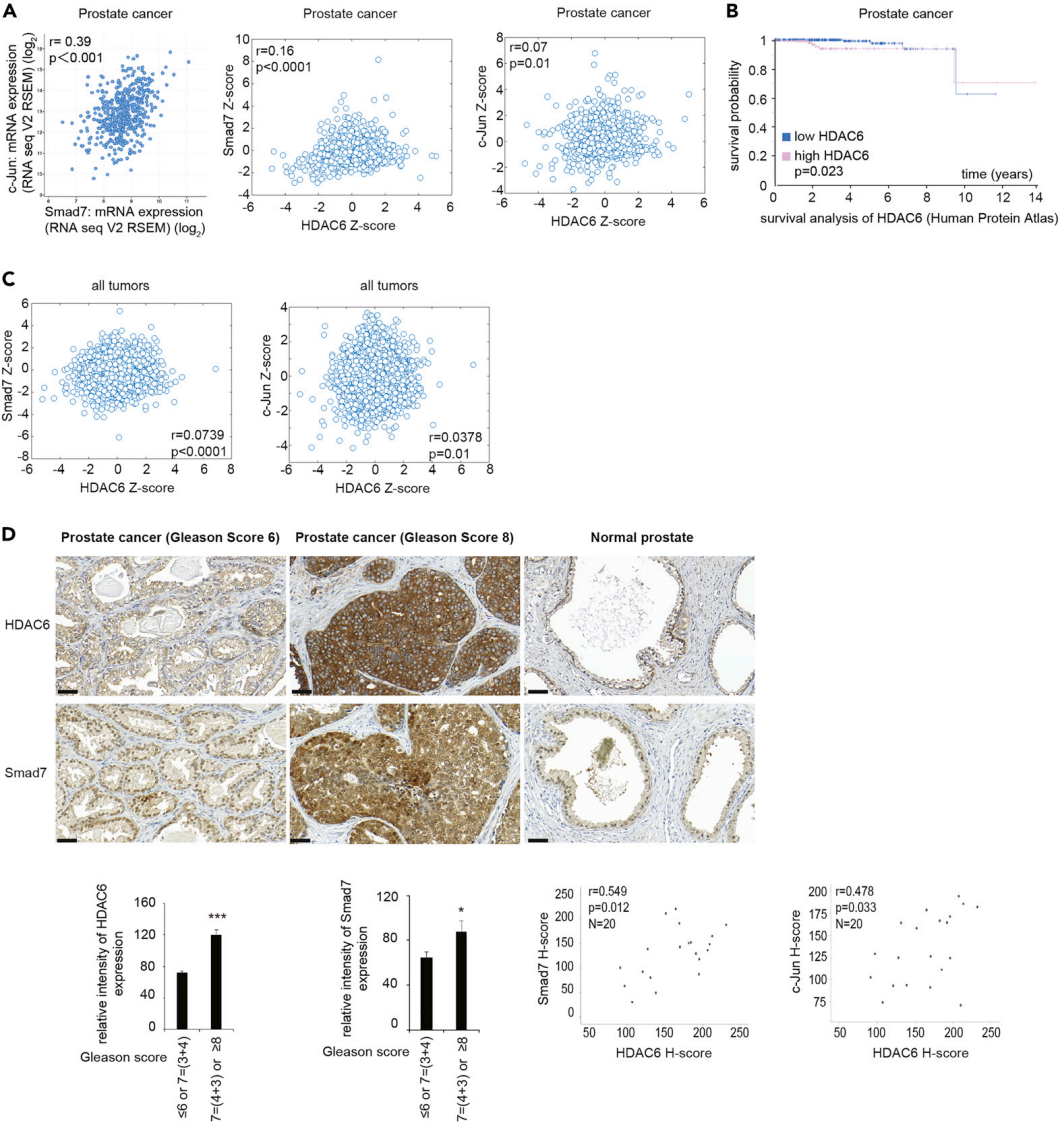
High Expression of *Smad7, c-Jun*, and *HDAC6* Is Correlated with Poor Prognosis in Patients with Prostate Cancer (A) The dot plots show positive correlations between expression of mRNA of *Smad7* and *c*-*Jun*, *Smad7* and *HDAC6;* and *Jun* and *HDAC6* in prostate cancer. Data were obtained from cBioPortal TCGA PanCancer Atlas databases in which log2 fold change (RNA seq V2 RSEM) and *Z* score are shown. p Value-Bootstrap hypothesis and Pearson correlation coefficient (r) are presented. (B) Kaplan Meier plot showing the survival probability of patients with prostate cancer categorized based on high and low expression of *HDAC6* mRNA. Representative image obtained from Human Protein Atlas. (C) The dot plots show positive correlations between expression of mRNA of *Smad7* and *HDAC6*; and *Jun* and *HDAC6* in all forms of cancer. (D) Prostate tumor tissue samples were stained with HDAC6, Smad7, and c-Jun antibodies. Quantification shows the means ± SEM of ten patients in each group. Mann-Whitney *U* test was used. ∗p < 0.05, ∗∗∗p < 0.001. Scale bar, 50 μm. The scatterplots show the positive correlations between Smad7 and HDAC6 protein, c-Jun, and HDAC6 proteins, respectively, in prostate cancer tissues. r = Pearson's coefficient test. See also Figure S4.

To investigate the clinical relevance of our findings, we used the cBioPortal database from TCGA. We found a positive correlation between *Smad7* and *c-Jun* mRNA, *Smad7* and *HDAC6* mRNA, and *c-Jun* and *HDAC6* mRNA (Figure 9A) in tumor material from patients with prostate cancer. Notably, high expression of *HDAC6* mRNA correlated with poor prognosis for patients with prostate cancer (Figure 9B), whereas no significant correlation between *Smad7* mRNA expression or *c-Jun* mRNA expression and poor prognosis for patients with prostate cancer was observed (data not shown). Moreover, we also observed a positive correlation between *Smad7* and *HDAC6* mRNA, and *c-Jun* and *HDAC6* mRNA in several various cancer forms (Figure 9C). The expression of HDAC6, Smad7, and c-Jun was examined by immunohistochemistry in tissue sections from patients with prostate cancer and in tissue sections from normal prostate gland. A significantly increased expression of HDAC6, Smad7, and c-Jun was observed in prostate cancer tissues with Gleason score ≥8 (including Gleason score 4 + 3) as compared with lower Gleason score ≤6 or 7 (including Gleason score 3 + 4), demonstrating that high expression of these proteins is correlated with prostate cancer progression. Moreover, a high co-expression of Smad7 and HDAC6, and c-Jun and HDAC6 was also observed in prostate cancer tissues, which correlated with poor prognosis (Figure 9D). The expression of both HDAC6 and Smad7 was found in basal epithelial cells in the normal prostate gland (Figure 9D). Smad7 expression has been reported to be localized in basal epithelial cells in the prostate gland in a murine prostate cancer model (Brodin et al., 1999), in line with our current observations. From these data, we conclude that the expression of HDAC6, Smad7, and c-Jun correlates with poor prognosis for patients with prostate cancer. Notably, a correlation between expression of *Smad7* mRNA and both *c-Jun* and *HDAC6* mRNA can also be seen in clinical material from patients with colorectal carcinoma, and high levels of *HDAC6* mRNA correlate with poor survival (Figure S4).

## Discussion

We report here that Smad7, a classical TGF-β target gene so far recognized mainly as a negative regulator of TGF-β signaling, also can act in the nucleus as a positive regulator of transcription of two tumor-promoting genes, which are linked to tumor invasion. We show herein that Smad7 binds to the regulatory regions of the *HDAC6* and *c-Jun* genes thereby promoting their expression in response to TGF-β. Using Hep3B cells, in which Smad7 is predominantly located in the nuclei regardless of TGF-β stimulation, Zhang et al. (2007) previously showed that forced expression of Smad7 repressed the transcriptional activity of TGF-β, suggesting that the effect of Smad7 on transcription varies between different cell lines. It is possible that the interaction between Smad7 and various partners differ in a contextual and kinetic manner after TGF-β stimulation, which can explain why Smad7 can act both as a positive and a negative regulator. The level of Smad7 may also be controlled in a contextual manner. For instance, Smad7 interacts with p300 via its MH2 domain leading to the acetylation by p300 at K64 and K70 in the N terminus of Smad7 (Gronroos et al., 2002). Acetylation at these lysines prevents the ubiquitination and degradation of Smad7. Smad7 also interacts, via its MH2 domain, with transcriptional repressors, such as HDACs, promoting its deacetylation, ubiquitination, and degradation. It has been reported that the other I-Smad, Smad6, forms a complex with phosphorylated Smad1, thereby inhibiting BMP signaling by disrupting the formation of a functional R-Smad-co-Smad complex. Smad6 acts as a transcriptional repressor by interacting with Hoxc-8 or by binding to DNA and recruiting transcriptional repressors like HDACs or CtBP to inhibit the transcription of target genes (Bai and Cao, 2002).

TGF-β induces the expression of Smad7 in the murine interleukin-2 (IL-2)-dependent T-lymphoma cell line CTLL2, which has high expression of the transcription factor ZEB1 and low or no expression of Smad7. In these cells, Smad7 binds to Smad responsive elements (SREs) upon TGF-β stimulation (Nakahata et al., 2010). A complex consisting of ZEB1, Smad7, R-Smads, and p300 is recruited to the SRE, leading to the activation of TGF-β target genes, including *Pai1* and *p21*. This study, showing a role for Smad7 in a transcriptionally active complex leading to enhanced TGF-β signaling, supports our findings that Smad7 binds to DNA and may also promote the binding of R-Smads to specific regions of DNA.

Using ChIP and DNAP, we mapped the binding site for Smad7 to 5′-GGCA-3′and 5′-GCCA-3′ motifs in the regulatory region of *HDAC6* and *c-Jun*, respectively. The Smad7-binding sites are adjacent to the Smad3 and Smad4 binding motif (5′-AGAC-3′). Zhang et al. (2007) previously reported that Smad7 binds to DNA directly via its MH2 domain. In contrast, our results suggest that K102 in the N-terminal region of Smad7 is important for binding to the *HDAC6* and *c-Jun* regulatory regions. The effect of Smad7 on transcription might be facilitated by the MH2-domain, which is known to bind to the transcriptional coregulator p300. The identification of Smad7 as a part of a transcriptional complex together with Smad2, 3, and 4 is particularly interesting in relation to the fact that Smads have low affinity for binding to DNA. Future studies will answer the question whether Smad7 promotes the regulation of other specific target genes besides *c-Jun* and *HDAC6*, in response to growth factor stimulation of cells. Furthermore, studies of the relation between the identified Smad7-binding sites and the recently identified additional binding motif for both TGF-β and BMP Smads, i.e., GGC(GC)|(CG) (Martin-Malpartida et al., 2017), will also be of importance to better understand the contextual regulation of gene expression in response to TGF-β stimulation of cells.

We have previously reported that TGF-β activates TRAF6 in PC3U cells causing activation of the p38 MAPK pathway leading to apoptosis (Sorrentino et al., 2008) and subsequent activation of c-Jun, promoting migration and invasion (Thakur et al., 2014). In this pathway, Smad7 acts as an adaptor protein facilitating TGF-β-induced activation of the JNK and p38-MAPK pathways (Sorrentino et al., 2008). These findings, together with the observations in the present study, suggest that Smad7 can exert its protumorigenic functions both as an effector of activation of non-canonical TRAF6-mediated responses and by regulation of gene transcription, in a manner dependent on its subcellular localization.

Smad7 has previously been found to be amplified in colorectal carcinoma, and this event is linked to poor prognosis for these patients (Boulay et al., 2003). Moreover, knockdown of Smad7 expression in a human colorectal carcinoma xenograft model has been shown to reduce tumor growth *in vivo* (Stolfi et al., 2014), giving further evidence for a tumor-promoting role of Smad7 in colorectal cancer. However, other studies have reported a dual role of Smad7 expression in colorectal cancer, in line with the dual role of TGF-β in other cancer types (Stolfi et al., 2013). In the current study, we report that Smad7 promotes expression of *HDAC6* in prostate cancer cells. High expression of *HDAC6* is significantly linked to poor prognosis for the patients with prostate cancer (Figure 9D), as well as colorectal cancer (Figure S4). Moreover, a high co-expression of Smad7 and HDAC6, and c-Jun and HDAC6, was observed in prostate cancer tissues, which correlated with poor prognosis (Figure 9D). We have recently reported that both TβRI and c-Jun are expressed at high levels in aggressive prostate cancer tissues (Gudey et al., 2017), and high levels of Smad7 have been reported to be expressed in prostate cancer bone metastases (Nordstrand et al., 2018), providing support for the notion that active TGF-β signaling promotes prostate cancer progression and metastases, in line with our observations in this report.

Histone deacetylase 6 (HDAC6) is a unique isoform of the HDAC family, which is overexpressed in some cancers, such as bladder cancer, malignant melanoma, and lung cancer (Li et al., 2018). Since HDAC6 regulates cell proliferation, metastasis, invasion, and mitosis in tumors (Lafarga et al., 2012; Lee et al., 2008; Wickstrom et al., 2010), several isoform-specific inhibitors of the enzymatic activity of HDAC6 have been developed and analyzed in clinical trials (Chuang et al., 2013; Seidel et al., 2016; Wu et al., 2018). We identified previously that c-Jun, a member of AP-1 transcription factors, is activated by TGF-β in a TRAF6-dependent manner leading to invasion of prostate cancer cells (Thakur et al., 2014) and that the Smad7-APC complex links TβRI to the microtubule system to promote migratory responses of prostate cancer cells (Ekman et al., 2012).

The fact that HDAC6 promotes migration and metastasis of cancer cells has made HDAC6 an interesting cancer drug target (Dallavalle et al., 2012). HDAC6 has unique features among HDAC isoforms in terms of its cytoplasmic localization and non-histone substrates. Since pan-HDAC inhibitors show a number of toxic effects, selective HDAC6 inhibitors, which have fewer side effects, may therefore be a preferred therapeutic option (Li et al., 2018). Anti-tumor effects of HDAC6-specific inhibitors on prostate cancer cells have been observed (Chuang et al., 2013; Seidel et al., 2016; Wu et al., 2018); however, the role of TGF-β and its connection to HDAC6 in prostate cancer progression have not been addressed before. In this study, we find that HDAC6 is acting downstream of Smad7 as overexpression of HDAC6 in Smad7-deficient MEFs increased c-Jun expression and their invasive capability (Figure 7). Moreover, treatment of prostate cancer cells with tubacin, an HDAC6 inhibitor, in a dose-dependent manner completely inhibited TGF-β-induced expression of *c-Jun* mRNA, as well as migration and invasion (Figures 8, S1C, S1D, S1G, and S1H), giving further experimental evidence for the important role for HDAC6 in TGF-β-induced migration and invasiveness of cancer cells and support for the potential usefulness of HDAC6 inhibitors for patients with aggressive prostate cancer. Our identification of HDAC6 as a downstream target gene of Smad7 in response to TGF-β is therefore of clinical importance for patients with prostate cancer, as HDAC6-inhibitors are currently investigated for their effects on cancer cells in clinical trials (Wang et al., 2018).

In summary, we have described a novel role for Smad7 as a transcriptional regulator of *c-Jun* and *HDAC6* in prostate cancer cells, promoting migration and invasion. Smad7 was shown to promote recruitment of R-Smad-Smad4 complexes to DNA, thus presenting a previously unknown function for Smad7. The TGF-β- and Smad7-induced increases of c-Jun and HDAC6 promote invasion of prostate cancer cells, making it relevant to investigate expression of Smad7, c-Jun, and HDAC6 in clinical tissue samples of patients with prostate cancer as potential biomarkers and drug targets.

### Limitation of the Study

The present study does not contain any investigation of the findings in an animal model. For instance, a mouse study in which the growth and invasion of prostate cancer cells, with or without knockdown of Smad7, HDAC6, or c-Jun, is missing.

### Resource Availability

#### Lead Contact

Further information and requests for resources and reagents should be directed to and will be fulfilled by the Lead Contact, Marene Landström (Marene.Landstrom@umu.se).

#### Materials Availability

We generated Smad7-mutant plasmids for this study as described in this article; contact Lead Contact (as described above).

#### Data and Code Availability

Public databases were used in our study as described in Transparent Methods.

## Methods

All methods can be found in the accompanying Transparent Methods supplemental file.

## Supplemental Information

Document S1. Transparent Methods and Figures S1–S4

Figure360. An Author Presentation of Graphical Abstract
