# Supplementary material for: Smad7 Enhances TGF-β-Induced Transcription of c-Jun and HDAC6 Promoting Invasion of Prostate Cancer Cells
Source: iScience. 2020 Sep 3;23(9):101470. doi: 10.1016/j.isci.2020.101470 (PMC7520897; doi:10.1016/j.isci.2020.101470)
Supplement: Document S1. Transparent Methods and Figures S1–S4 [file mmc1.pdf]

iScience, Volume *23*

Supplemental Information

Smad7 Enhances TGF-β-Induced Transcription of c-Jun and HDAC6 Promoting Invasion of Prostate Cancer Cells

Noopur Thakur, Anahita Hamidi, Jie Song, Susumu Itoh, Anders Bergh, Carl-Henrik Heldin, and Maréne Landström

Supplemental Items

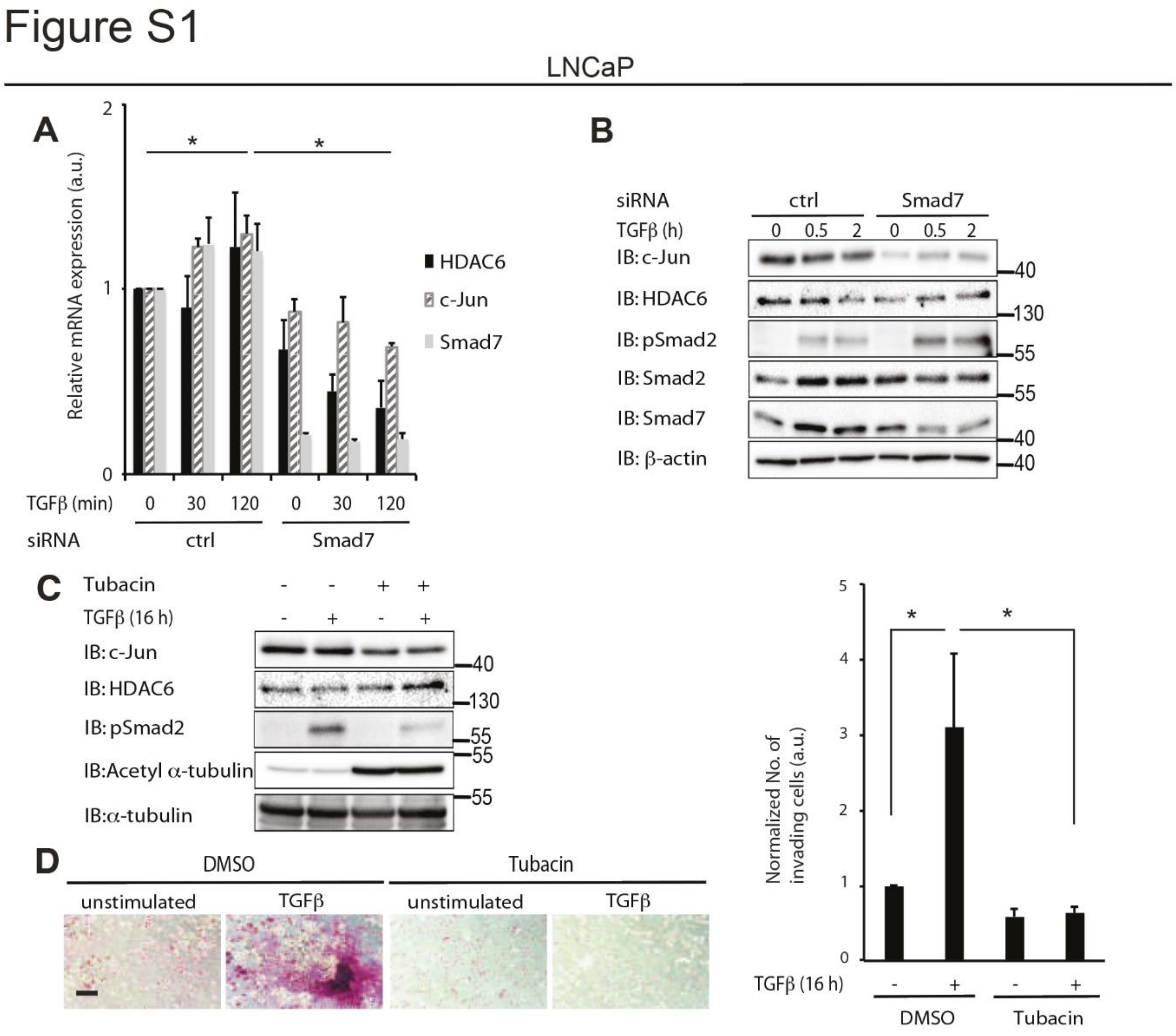

Figure S1. TGFβ-induced c-Jun-mediated invasion of prostate cancer cells is dependent on Smad7 and HDAC6, related to Figure 1. LNCaP **(A, B)** cells transfected with control or Smad7 siRNA and stimulated with TGFβ or not, were lysed and subjected to RT-PCR using primers for c-Jun, HDAC6 or Smad7 (**A**), or to IB for c-Jun, HDAC6, Smad2, pSmad2, Smad7 and β-actin (**B**). LNCaP **(C)** cells were treated or not with 2.5 μM tubacin prior to TGFβ stimulation and subjected to IB for c-Jun, HDAC6, pSmad2, acetyl α-tubulin and α-tubulin. LNCaP **(D)** cells were subjected to an invasion assay for 16 h, without or with TGFβ stimulation and without or with tubacin. Scale bar, 100 μM. Bar graph are means ± SEM from three independent experiments. One-way ANOVA was used as the statistical test. $*P < 0.05$

Figure S2

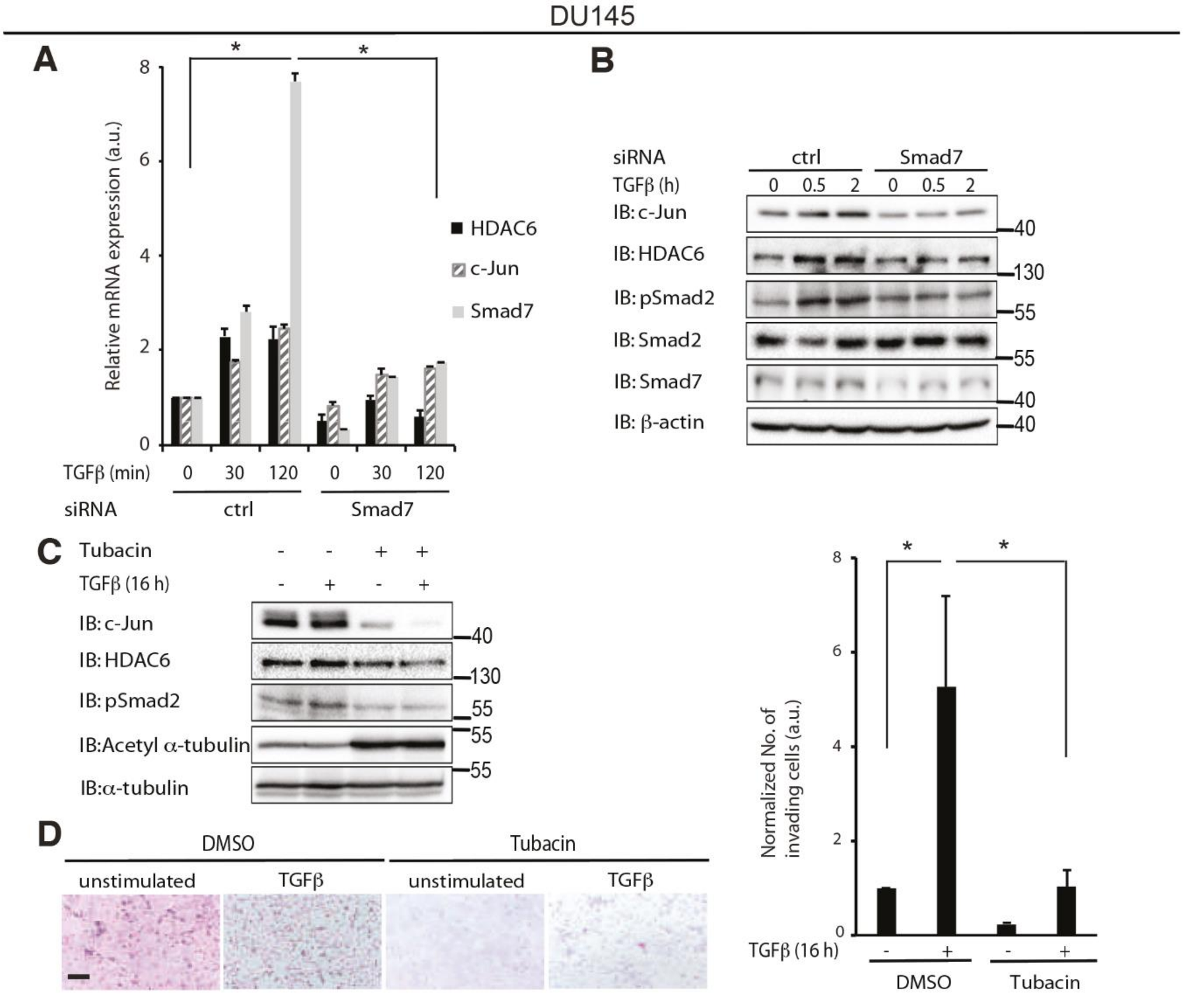

Figure S2. TGFβ-induced c-Jun-mediated invasion of prostate cancer cells is dependent on Smad7 and HDAC6, related to Figure 1. DU145 cells **(A, B)** transfected with control or Smad7 siRNA and stimulated with TGFβ or not, were lysed and subjected to RT-PCR using primers for c-Jun, HDAC6 or Smad7 (**A**), or to IB for c-Jun, HDAC6, Smad2, pSmad2, Smad7 and β-actin (**B**). DU145 **(C)** cells were treated or not with 2.5 μM tubacin prior to TGFβ stimulation and subjected to IB for c-Jun, HDAC6, pSmad2, acetyl α-tubulin and α-tubulin. DU145 **(D)** cells were subjected to an invasion assay for 16 h, without or with TGFβ stimulation and without or with tubacin. Scale bar, 100 μM. Bar graph are means ± SEM from three independent experiments. One-way ANOVA was used as the statistical test. $*P < 0.05$

Figure S3

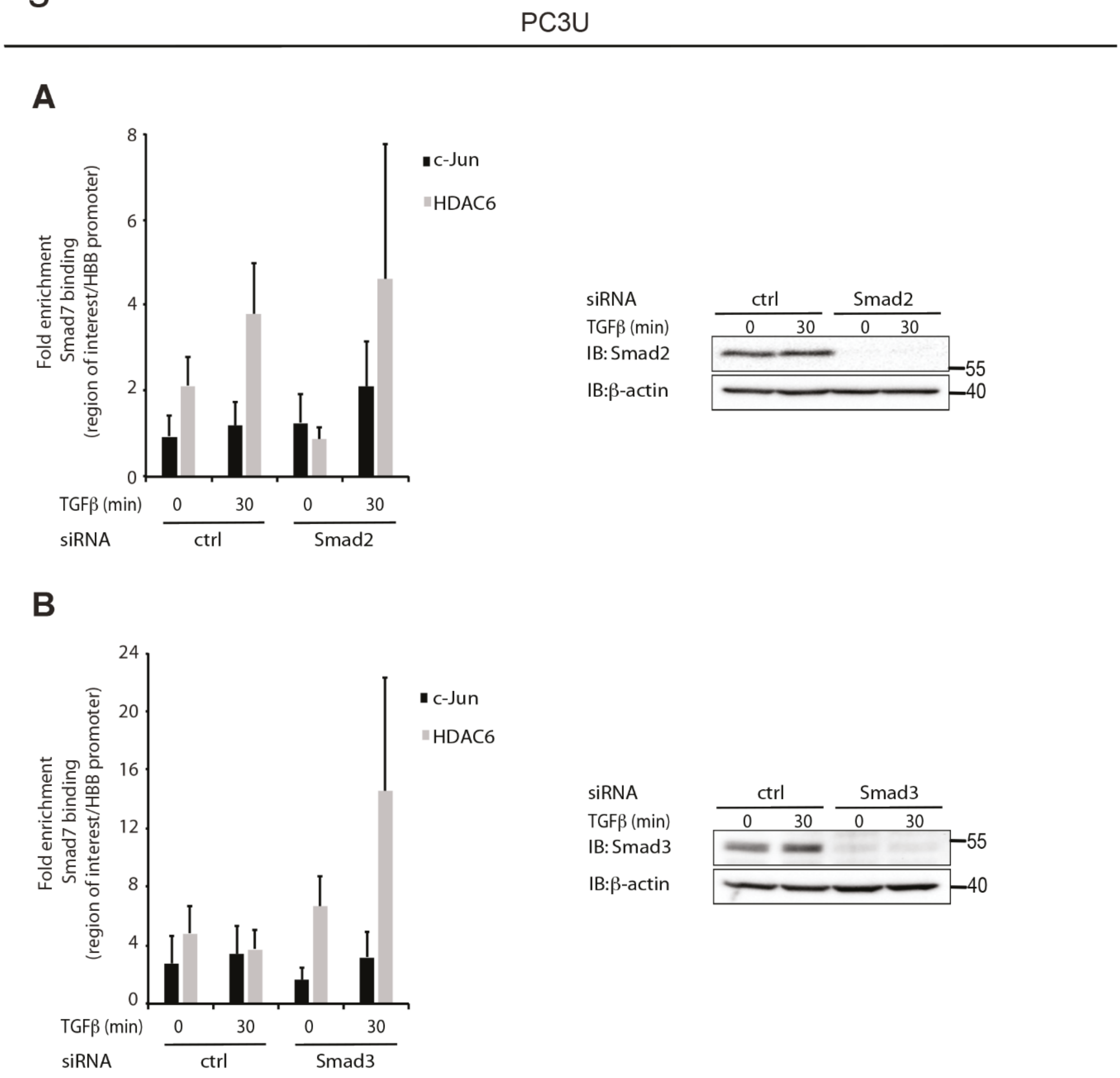

Figure S3. Smad7 binding to DNA is not dependent on Smad2 and 3, related to Figure 3. Lysates from PC3U cells transfected with ctrl, Smad2 **(A)** or Smad3 **(B)** siRNA, and treated or not with TGFβ for 30 min, were subjected to ChIP with a Smad7 antibody and RT-PCR with primers recognizing *c-Jun* promoter, *HDAC6* regulatory region or hemoglobin subunit β (HBB) promoter. Graphs are means ± SEM from three independent experiments. One-way ANOVA was used as the statistical test. Control IB to check the knock-down efficiency is shown.

Figure S4

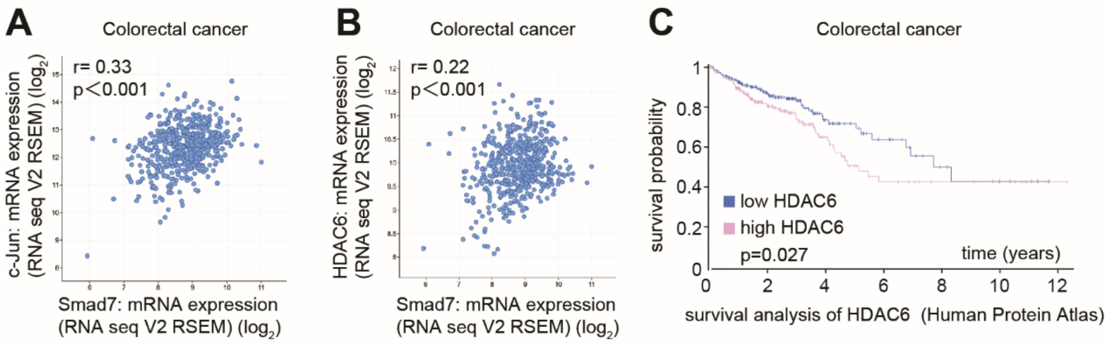

Figure S4. *Smad7* mRNA expression correlates with *c-Jun* and *HDAC6* mRNA expression, as well as with poor survival in colorectal cancer, related to Figure 9. (A-B) The dot plots show the positively correlation between mRNA expression of Smad7 and c-Jun **(A)**, HDAC6 **(B)** in the colorectal cancer. Data obtained from cBioPortal TCGA PanCancer Atlas databases in which log2 fold change (RNA seq V2 RSEM) was represented. *P* value-Bootstrap hypothesis and Pearson correlation coefficient (r) were represented. **(C)** Kaplan Meier Plot showing the survival probability of patients with colorectal cancer categorized based on high and low expression of HDAC6 mRNA. Representative image obtained from Human Protein Atlas.

Transparent Methods

Cell culture

The human prostate cancer cell lines PC3U, a sub-line originating from PC3 cells (Franzen et al., 1993), and LNCaP were cultured in RPMI 1640 medium supplemented with L-glutamine, whereas DU145 cells were cultured in Minimum Essential Medium, and wild-type (wt) and Smad7$^{-/-}$ mouse embryo fibroblasts (MEFs) were grown in Dulbecco´s modified Eagle Medium (DMEM); in each case, media were supplemented with 10% fetal bovine serum (FBS) and penicillin/streptomycin. Cells were cultured at 37 °C in an atmosphere of 5% CO_2. Transient transfections of PC3U cells and of wt and Smad7$^{-/-}$ MEFs were performed as described earlier (Sorrentino et al., 2008).

Antibodies and other reagents

TGFβ1 was purchased from R&D Systems, MN, USA. Antibodies against c-Jun, phospho (p)-Ser63 c-Jun, Smad2/3, Smad4 and Lamin A/C were from Cell Signaling, MA, USA. Antibodies against β-actin, α-tubulin and α-tubulin were from Sigma, MI, USA. Smad7 (used in ChIP assays), acetyl α-tubulin and HDAC6 antibodies were from Santa Cruz Biotechnology, TX, USA. Smad7 antibody (used for Western blotting) was purchased from R&D Systems, Inc, MN, USA. Smad2 antibody was purchased from Abcam, Cambridge, UK. Phospho-Smad2 antiserum was generated in house. Secondary HRP conjugated anti-mouse, anti-rabbit and anti-goat IgG were from GE Healthcare, Uppsala, Sweden. Tubacin was purchased from Selleckchem, TX, USA. Fugene HD transfection reagent (for transfection of PC3U; LNCaP and DU145 cells) was from Promega Corporation, WI, USA. Lipofectamine 3000 transfection reagent (for transfection of MEF cells) was from ThermoFisher Scientific, MA, USA.

Preparation of total RNA and cDNA

Total RNA was isolated using an RNeasy mini kit (Qiagen), according to the manufacturer's instructions. The isolated RNA was quantified using a Nanodrop ND-100 spectrophotometer. Two micrograms of total RNA were used for cDNA preparation. For cDNA synthesis, Thermoscript RT PCR system (Invitrogen) was used, following the manufacturer's instructions. The purity of cDNA obtained was determined by using a Nanodrop spectrophotometer. After quantification, the cDNA was diluted 10-fold with RNase-free water.

Analysis of mRNA levels by quantitative Real Time PCR (qRT-PCR)

cDNA purified from the cells was amplified and measured in duplicates with RT-PCR using Stratagene system, with SYBR green (Applied Biosystems) to detect the PCR product. Specific primers for HDAC6, c-Jun and Smad7 were constructed with the aid of the Primer3plus free software http://www.bioinformatics.nl/cgiin/primer3plus/primer3plus.cgi and the primers were purchased from Sigma Aldrich. The primer sequences were: Smad7, forward primer (FP), TCCTGCTGTGCAAAGTGTTC, reverse primer (RP), TCTGGACAGTCTGCAGTTGG; human HDAC6, FP, TATCTGCCCCAGTACCTTCG, RP, GGACATCCCAATCCACAATC; mouse HDAC6, FP, TCCACCGGCCAAGATTCTTC, RP, GCCTTTCTTCTTTACCTCCGCT; human GAPDH, FP, GAAGATGGTG ATGGGATTTT C, RP, GAAGGTGAAG GTCGGAGT; mouse GAPDH, FP, TGTGTCCGTCGTGGATCTGA, RP, CCTGCTTCACCACCTTCTTGA ; c-Jun, FP, CCCCAAGATC CTGAAACAGA, RP, CCGTTGCTGG ACTGGATTAT; mouse Smad7, FP, CTGGTGTGCTGCAACCCCCATC, RP, ATCTGGACAGCCTGCAGTTGGTT. A reaction mixture containing 12.5 µl of SYBR green PCR mix, 2 µl of diluted cDNA, 10 pmoles per µl of forward and reverse primers and RNase-free water, was used (in a total volume of 25 µl). The *GAPDH* gene was used as an internal reference in the real-time PCR protocol. As a negative control, one sample with no cDNA (only with RNase-free water) was included in each run of the RT-PCR assay, for each primer pair.

Immunoblotting and *in vivo* protein interactions

PC3U, LNCaP and DU145 cells were grown in 10-cm dishes and starved for 12–18 h in medium containing 1% FBS, glutamine and penicillin/streptomycin. Wild-type and Smad7$^{-/-}$ MEFs were grown in 10-cm dishes and starved for at least 12 h in serum-free medium. The cells were then treated with 5 ng/ml TGFβ for the indicated time periods, and then harvested for subsequent analysis. Equal amounts of proteins were subjected to immunoprecipitation, followed by Sodium dodecylsulfate polyacrylamide electrophoresis in 8, 10 or 12% polyacrylamide gels, followed by immunoblotting using polyvinylidene difluoride membranes, as described previously (Sorrentino et al., 2008).

DNAP assays

PC3U cells, grown in 10-cm dishes for 60–72 h to 50% confluency, were stimulated with 5 ng/ml TGFβ for different time periods. Proteins extracted in NP-40 lysis buffer (1% NP-40, 150 mM NaCl, 50 mM Tris, pH 8.0), were incubated with double-stranded biotinylated oligonucleotide probes from the *c-Jun* or *HDAC6* regulatory regions, or no probe, in the presence of 5 μg salmon sperm DNA for 2 h at4°C, followed by incubation for 45 min with streptavidin-beads and centrifugation. The DNA-bound protein complexes were washed four times in NP-40 lysis buffer and then resolved by SDS-PAGE followed by immunoblotting.
The following oligos were used: HDAC6, forward oligo (FO), CGGGGGCTCATTGCTCCGTGAAAG**GGCAAGAC**CAGGGAAAGAGAATCGTGTA, reverse oligo (RO), TACACGATTCTCTTTCCCTGGTCTTGCCCTTTCACGGAGCAATGAGCCCCCG;
c-Jun, FO, GTCGGAGTCCGGGCG**GCCAAGAC**CCGCCGCCGGCCGGCCGGCCACTGCAGGGTCCGCAC, RO, GTGCGGACCCTGCAGTGGCCGGCCGGCCGGCGGCGGTGGCGTCTCGCCCGGACTCCGAC.

Nuclear and cytoplasmic fractionation assay
PC3U cells were grown in 10-cm dishes and starved for 12 – 18 h in medium containing 1% FBS, L-glutamine and penicillin/streptomycin. The starved cells were then treated with TGFβ for the indicated time periods, and the cells were washed two times in ice-cold PBS, then ice-cold lysis buffer (1% Trition X-100, 10 mM MES pH 6.2, 10 mM NaCl, 1.5 mM $MgCl_2$, 1 mM EDTA, and protease and phosphatase inhibitors) was added. After 15 min incubation on ice, cells were scraped and centrifuged; the supernatant was collected as the cytoplasmic fraction. The pellet was washed in wash buffer (10 mM MES pH 6.2, 10 mM NaCl, 1.5 mM $MgCl_2$, 1 mM EDTA) and then re-suspended in nuclear extraction buffer (0.5% Triton X-100, 25 mM Tris-HCl pH 7.5, 1 mM EDTA, 0.5 M NaCl, and protease and phosphatase inhibitors) and incubated on ice for 20 min; after centrifugation, the supernatant was collected as the nuclear protein fraction. The protein concentration of samples was measured using the BCA assay (Thermo Scientific #23227) and equal quantity of samples were loaded on the gel. As markers for nuclear and cytoplasmic fractions, immunoblotting with lamin A/C rabbit antibody (Cell Signaling 2032) and β-tubulin mouse antibody (Sigma), respectively, were used.

siRNA transfection
PC3U, LNCaP and DU145 cells were transfected with ON-TARGET plus SMART pool Human Smad7 siRNA (L-020068-00), ON-TARGET plus SMART pool Human HDAC6 siRNA (L-003499-00), ON-TARGET plus SMART pool Human Smad2 siRNA (L-003561-00), ON-TARGET plus SMART pool Human Smad3 siRNA (L-020067-00), ON-TARGET plus SMART pool Human Smad4 siRNA (L-003902-00) or ON-TARGET plus Non-targeting pool (D-001810-10-20) as a negative control, using Dharmafect 2 as transfection reagent (Dharmacon, Inc, IL, USA). The siRNA and Dharmafect 2 were mixed in separate tubes and incubated for 5 minutes. They were then mixed, and incubated for another 30 minutes, and then added to the cells grown in 10-cm plates; cell lysates were then analyzed by SDS-PAGE and immunoblotting.

Chromatin immunoprecipitation
Five biological replicates of each chromatin immunoprecipitation (ChIP) were performed according to the protocol provided by Abcam using the Smad7 N-19 Goat antibody (Santa Cruz Biotechnology). After purification, the ChIP DNA was amplified by PCR in triplicate with the following primers: HDAC6 ChIP forward primer (FP), AGAGTAGAAGGGGCGGTGAT, reverse primer (RP), CTCCACAGCCTTCCAAACTC; c-Jun ChIP FP, CATTACCTCATCCCGTGAGC, RP, GCCCGAGCTCAACACTTATC.

Invasion assay
Cell invasion was determined using the BD BioCoat Growth Factor Reduced MATRIGEL Invasion chamber according to the manufacturer's protocol. After 16 hours with or without TGFβ, cells on the upper side of the membrane were scraped off and cells which had invaded to the lower side of the membrane were fixed in 4% formaldehyde and stained by Giemsa and photographed using a Zeiss Axiovert 40CFL microscope. Primary images were acquired using the Zen program. The number of invading cells was quantified by measuring the number of pixels using Photoshop 6.0 (Adobe). Pictures shown are representative images from three different experiments.

Scratch wound healing assay
PC3U and MEF cells were cultured in 6-well plates. Twenty four hours later, cells were transfected with siRNA or not, and after another 24 h the cells were treated or not with inhibitor. TGFβ was added to the cells after one hour and "wounds" were made using a 200-μl pipette tip. Pictures of the "wounds" were taken immediately and 24 h later using Zeiss Axiovert 40CFL microscope. Primary images were acquired using the Axiovision program and analyzed by the Tscratch program. The percentage of the open wound was calculated by dividing the area of

the gap after 24 h by that at 0 h. Then, the percentage of the wound closure was calculated by subtracting the open wound percentage from 100. Pictures shown are representative images from three different experiments.

Immunohistochemistry

The tissue slides were deparaffinized in xylene, rehydrated through graded alcohols, and incubated for antigen retrieval in the Retriever 2100 (Proteogenix). After washing in running water, the slides were incubated in 3% hydrogen peroxide in methanol for 10 minutes to block endogenous peroxidase. After incubation in 5% goat serum for 30 minutes, the sections were incubated overnight at 4°C in primary antibody diluted in 5% goat serum. HDAC6 antibody (Sigma), Smad7 antibody (R&D) and c-Jun antibody (Atlas antibodies) were used at a 1:100 dilution. After washing with PBS, the slides were incubated with Real EnVision detection System (Dako). The reaction was developed under microscopic control and then stopped with tap water. The sections were stained with hematoxylin, dehydrated, and mounted. Images were acquired with Pannoramic 250 Flash. The quantification of immunohistochemistry was performed by software ImageJ as described before (Detre et al., 1995). In briefly, staining intensity was grouped into 4 levels (0, negative; 1, weak staining; 2, moderate staining; and 3, strong staining). The number of cells in each group were counted, as well as the total number of the cells. IHA H-score=(percentage of cells in group1 *1) + (percentage of cells in group2 *2) + (percentage of cells in group3 *3). An ethical permit to use tumour tissues for generation of tissue slides was provided by the Umeå Ethical Review Board in full agreement with the Swedish Ethical Review Act (540/03, Dnr 03–482).

In silico gene expression analysis-correlation analysis between Smad7 and c-Jun; Smad7 and HDAC6; c-Jun and HDAC6

Data on the expression of *c-Jun*, *Smad7*, and *HDAC6* mRNA in colorectal and prostate cancer and different cancer forms was obtained from cBioPortal TCGA PanCancer Atlas databases. Specifically, we used gene expression data for prostate cancer from the five different datasets as described below:

1. Prostate adenocarcinoma from TCGA (498 samples)
2. Metastatic prostate adenocarcinoma from PNAS 2019 (212 samples)
3. Neuroendocrine prostate adenocarcinoma from Nat Med 2016 (49 samples)
4. Prostate adenocarcinoma from Fred Hutchinson (Nat Med 2016, 171 samples)
5. Prostate adenocarcinoma from MSKCC (Cancer Cell 2010, 150 samples)

We then correlated the expression of *c-Jun* and *Smad7*, *Smad7* and *HDAC6*; and *HDAC6* and *c-Jun* in colorectal and prostate cancer.

For each dataset (1-5), we performed two different analyses: first, we observe direct correlation between target gene expression (log2-transformed), and we estimated the significance of correlation using Pearson and Spearman tests. Secondly, we extracted the specific samples that have a Z-value above 2 (overexpressed) or under -2 (repressed) for each target gene compared to its general expression in the set. Then, we estimated the log2-odds ratio for the co-presence of two target genes with overexpression or repression in the same sample, in order to calculate either the co-occurrence or the mutual exclusivity for the presence of alterations in a pair of genes.

As a result, we observe a significant gene expression correlation in three datasets out of five for HDAC6 and JUN (and set #3 might have an artifact in some samples that affect the correlation), while only one dataset showed correlation between HDAC6 and SMAD7 (in this case, it also showed tendency to co-occurrence for the alterations of the same genes).

The combined results (meta-analysis) was done using the Z-Scores of each gene in each sample in order to have a common measure for all the experiments. Pearson Correlation and P-values were calculated from the combined datasets.

We obtained survival data from the TCGA study of prostate adenocarcinoma (494 samples) and human colorectal tumours (597 samples) reported in Human Protein Atlas, and analyzed the correlation between patient survival and mRNA expression levels of *HDAC6*. Survival analysis was performed by dividing the patients into two groups with low and high levels of mRNA expression. (Abida et al., 2019; Beltran et al., 2016; Kumar et al., 2016; Taylor et al., 2010; Weinstein, 2013).

Statistical analysis

Statistical analyses between all groups were performed using the analysis of variance (one-way ANOVA) test. F-test was used to derive *P* values. Values are expressed as mean ± SEM of three independent experiments (N=3). *P* values of < 0.05 were considered as statistically significant; * $P<0.05$, ** $P<0.01$, *** $P<0.001$.

Supplemental References

Abida, W., Cyrta, J., Heller, G., Prandi, D., Armenia, J., Coleman, I., Cieslik, M., Benelli, M., Robinson, D., Van Allen, E.M., *et al.* (2019). Genomic correlates of clinical outcome in advanced prostate cancer. Proc Natl Acad Sci U S A *116*, 11428-11436.

Beltran, H., Prandi, D., Mosquera, J.M., Benelli, M., Puca, L., Cyrta, J., Marotz, C., Giannopoulou, E., Chakravarthi, B.V., Varambally, S., *et al.* (2016). Divergent clonal evolution of castration-resistant neuroendocrine prostate cancer. Nat Med *22*, 298-305.

Detre, S., Saclani Jotti, G., and Dowsett, M. (1995). A "quickscore" method for immunohistochemical semiquantitation: validation for oestrogen receptor in breast carcinomas. J Clin Pathol *48*, 876-878.

Franzen, P., Ichijo, H., and Miyazono, K. (1993). Different signals mediate transforming growth factor-beta 1-induced growth inhibition and extracellular matrix production in prostatic carcinoma cells. Exp Cell Res *207*, 1-7.

Kumar, A., Coleman, I., Morrissey, C., Zhang, X., True, L.D., Gulati, R., Etzioni, R., Bolouri, H., Montgomery, B., White, T., *et al.* (2016). Substantial interindividual and limited intraindividual genomic diversity among tumors from men with metastatic prostate cancer. Nat Med *22*, 369-378.

Sorrentino, A., Thakur, N., Grimsby, S., Marcusson, A., von Bulow, V., Schuster, N., Zhang, S., Heldin, C.H., and Landstrom, M. (2008). The type I TGF-beta receptor engages TRAF6 to activate TAK1 in a receptor kinase-independent manner. Nat Cell Biol *10*, 1199-1207.

Taylor, B.S., Schultz, N., Hieronymus, H., Gopalan, A., Xiao, Y., Carver, B.S., Arora, V.K., Kaushik, P., Cerami, E., Reva, B., *et al.* (2010). Integrative genomic profiling of human prostate cancer. Cancer Cell *18*, 11-22.

Weinstein, J.N., Collisson, E.A., Miiis, G.B., Mills Shaw, K.R., Ozenberger, B.A., Ellrott, K., Shmulevich, I., Sander, C., Stuart, J.M., (2013). The Cancer Genome Atlas Pan-Cancer Analysis Project. Nat Genet *45*, 1113-1120.
